# Conotoxin MVIIA improves cell viability and antioxidant system after spinal cord injury in rats

**DOI:** 10.1371/journal.pone.0204948

**Published:** 2018-10-04

**Authors:** Karen M. Oliveira, Nancy S. Binda, Mário Sérgio L. Lavor, Carla M. O. Silva, Isabel R. Rosado, Endrigo L. A. Gabellini, Juliana F. Da Silva, Camila M. Oliveira, Marília M. Melo, Marcus Vinícius Gomez, Eliane G. Melo

**Affiliations:** 1 Clinical and Surgery Department, Veterinary School, Minas Gerais Federal University, *Campus* Pampulha, Belo Horizonte, Minas Gerais, Brazil; 2 Laboratory of Toxins, Institute of Education and Research, Santa Casa, Belo Horizonte, Minas Gerais, Brazil; 3 Department of Agrarian and Environmental Sciences, Santa Cruz State University, Ilhéus, Bahia, Brazil; 4 Veterinary Medicine Department, Uberaba University, Uberada, Minas Gerais, Brazil; 5 Medical School, Paraná Federal University, Paraná, Brazil; Universidad de Castilla-La Mancha, SPAIN

## Abstract

This study evaluates whether intrathecal MVIIA injection after spinal cord injury (SCI) elicits neuroprotective effects. The test rats were randomly distributed into six groups— sham, placebo, MVIIA 2.5 μM, MVIIA 5 μM, MVIIA 10 μM, and MVIIA 20 μM—and were administered the treatment four hours after SCI. After the optimal MVIIA dose (MVIIA 10 μM) was defined, the best time for application, one or four hours, was analyzed. Locomotor hind limb function and side effects were assessed. Forty-eight hours after the injury and immediately after euthanasia, spinal cord segments were removed from the test rats. Cell viability, reactive oxygen species, lipid peroxidation, and glutamate release were investigated. To examine the MVIIA mechanism of action, the gene expressions of pro-apoptotic (Bax, nNOS, and caspase-3, -8, -9, -12) and anti-apoptotic (Bcl-xl) factors in the spinal cord tissue samples were determined by real-time PCR, and the activities of antioxidant enzymes were also investigated. Application of intrathecal MVIIA 10 μM four hours after SCI prompted a neuroprotective effect: neuronal death decreased (22.46%), oxidative stress diminished, pro-apoptotic factors (Bax, nNOS, and caspase-3, -8) were expressed to a lesser extent, and mitochondrial viability as well as anti-apoptotic factor (Bcl-xl) expression increased. These results suggested that MVIIA provided neuroprotection through antioxidant effects. Indeed, superoxide dismutase (188.41%), and glutathione peroxidase (199.96%), reductase (193.86%), and transferase (175.93%) expressions increased. Therefore, intrathecal MVIIA (MVIIA 10 μM, 4 h) application has neuroprotective potential, and the possible mechanisms are related to antioxidant agent modulation and to intrinsic and extrinsic apoptotic pathways.

## Introduction

Spinal cord injury (SCI) is a serious event that can be devastating to the patient from both the economic and social standpoints. SCI constitutes one of the main rehabilitation challenges and is directly associated with permanent disabilities and reduced patient life expectancy [1–3]. Neurological deficits are related to the initial trauma and mainly to the extent of secondary neurodegenerative lesions (e.g., glutamate-mediated excitotoxicity, imbalance in calcium homeostasis and consequent mitochondrial dysfunction, reactive oxygen species (ROS) generation, lipid peroxidation (LP), and apoptosis [4–9]). Because the initial impact can only be prevented, therapeutic strategies regarding SCI have focused on the cascade of secondary events triggered soon after spinal trauma [1–2].

The toxin ω-conotoxin MVIIA (, designated MVIIA hereafter) was first obtained from the marine snail *Conus magus*. It is initially purified as SNX-111 and is classified as a potent reversible N-type calcium channel blocker [10–13]. In synaptosomes, MVIIA inhibits the release of various neurotransmitters, such as norepinephrine [14–17] and glutamate [18], which are essential to secondary spinal injury development [19–21]. The analgesic properties of MVIIA have led the US Food and Drug Administration to approve its therapeutic application [22]. MVIIA is currently used to treat severe chronic and neuropathic cancer pain [19, 22–26]. Additionally, MVIIA application elicits significant neuroprotection in rat traumatic brain injury [24, 27–30], in rat [31–33] and rabbit [34] cerebral ischemia models, and in rat SCI [35].

Given the peptidic nature of MVIIA, the cone snail toxins are not orally available and they must be delivered directly into the central nervous system (CNS) to avoid its degradation by proteolytic enzymes and the use of excessive MVIIA doses, which could lead to severe side effects. In a previous experiment [36], our group applied intralesional MVIIA directly on the target organ, which allowed targeted delivery of the optimal drug dose. However, MVIIA application five minutes after SCI did not promote neuroprotective effects. Bearing in mind that the potential MVIIA neuroprotective action has been reported for MVIIA application between 15 min [30] and 24 h [31–33] after global cerebral ischemia [31], focal cerebral ischemia [32], transient cerebral ischemia [37], and traumatic brain injury [30] in rats, we decided to apply different MVIIA doses at distinct administration times after acute SCI in rats, intrathecal administration, which is a more clinically viable strategy to deliver the drug directly to the affected site.

## Materials and methods

### Animals

Three-month-old adult male Wistar rats weighing between 250 and 310 g and provided by the Minas Gerais Federal University were housed in plastic boxes in groups of four, in a controlled environment (12:12 light/dark cycle; 22 ± 2 ^o^C; humidity 50 ± 5%); commercial rodent food and water were available *ad libitum*. All the rats were subjected to two-week acclimatization. This study was carried out in strict accordance with the recommendations published in the Guide for the Care and Use of Laboratory Animals of the National Institute of Health and was approved by the Ethics Committee on Animal Experimentation of the Minas Gerais Federal University (protocol number 226/2012).

### Spinal cord injury procedure

Twenty minutes before the SCI procedure, the rats received prophylactic antibiotic cephalotin (60 mg.kg^-1^, subcutaneous injection) and morphine sulfate (2.5 mg.kg^-1^, subcutaneous injection). Anesthesia was then induced and maintained with isoflurane in a non-rebreathing circuit, through a facemask. Hair was removed from the thoracic to the lumbar level with an electric shaver. Then, the rat’s back was disinfected with antiseptic solution consisting of povidone-iodine, followed by alcohol 70%. The rats were positioned in a stereotaxic apparatus [36, 38–41] and prepared for aseptic surgery. An incision was made in the dorsal midline skin and subcutaneous tissue extending from T8 to L1, and the muscle and tissue overlying the spinal column was blunt dissected away, to reveal the laminae. By using the T13 spiny process as landmark, T12 laminectomy was performed with a pneumatic drill, and the lamina was carefully removed, to expose the spinal cord. Extradural moderate compression of the spinal cord at the T12 vertebral level was conducted for five minutes as described previously [36, 38, 39]; a 40.5 g.cm^-1^ weight was employed. Sham-operated rats (SHAM rats) did not receive the compression. The incision was closed in two layers, with continuous simple pattern suture (non-absorbable suture material polypropylene 3-0) and with separate simple pattern suture (non-absorbable suture material polypropylene 3-0). The rats received fluidotherapy with saline solution (15 mL.kg^-1^, subcutaneous injection) and were allowed to recover from anesthesia in a warmed (37 ^o^C) box under oxygen therapy and with veterinary assistance. Post-operative care protocols to alleviate suffering in the animals involved administration of morphine sulfate (2.5 mg.kg^-1^, subcutaneous injection every 4 h) on the surgery day and of tramadol chloride (10 mg.kg^-1^, subcutaneous injection every 8 h) on the two days following SCI as well as cephalexin administration (60 mg.kg^-1^, oral administration, twice daily), control of water and food intake, and manual bladder expression (three times a day) until euthanasia were accomplished. The rats were evaluated on a daily basis and checked for pain and stress signals.

### Drug administration

MVIIA was dissolved in sterile PBS and centrifuged until full dissolution was achieved. Before use, the MVIIA solutions were stored as 20-μL working aliquots containing MVIIA at 50 pmol/μL, at -20°C.

**Fig 1:**
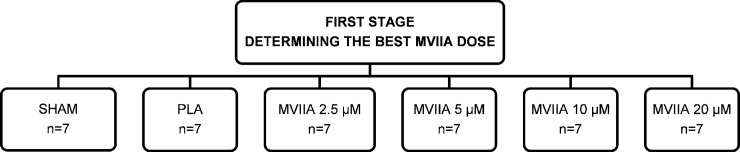
Flow chart of the study design to determine the best omega-conotoxin MVIIA dose in spinal cord injury. SHAM rats and rats subjected to SCI and injected with placebo (sterile PBS/vehicle, control, designated PLA rats) or MVIIA (designated MVIIA 2.5 μM rats, MVIIA 5 μM rats, MVIIA 10 μM rats, and MVIIA 20 μM rats).

To evaluate dose response, 42 rats were randomly distributed into six groups: SHAM rats and rats subjected to SCI and injected with placebo (sterile PBS/vehicle, control, designated PLA rats) or MVIIA (designated MVIIA 2.5 μM rats, MVIIA 5 μM rats, MVIIA 10 μM rats, and MVIIA 20 μM rats). The MVIIA 2.5 μM, MVIIA 5 μM, MVIIA 10 μM, and MVIIA 20 μM solutions were prepared in sterile PBS by diluting 25, 50, 100, and 200 pmol of MVIIA in a total volume of 10 μL, respectively. The PLA and MVIIA rats received the specific treatment via intrathecal route 4 h after SCI. Placebo or MVIIA solution was delivered by means of a 10-μL Hamilton needle, as previously described by Mestre et al. [42]. Covering the rats with a surgical compress helped to contain and to tranquilize them (Fig 1).

**Fig 2:**
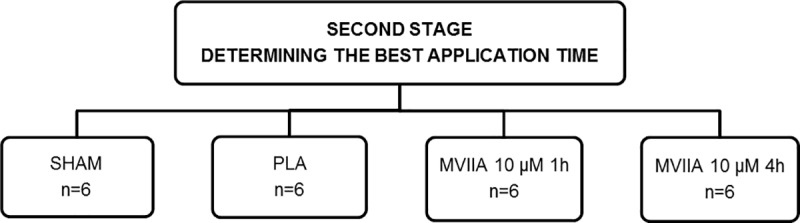
Flow chart of the study design to determine the best application time for omega-conotoxin MVIIA in spinal cord injury. SHAM rats and rats subjected to SCI and injected with placebo (sterile PBS/vehicle, control, designated PLA rats) or MVIIA (designated MVIIA 2.5 μM rats, MVIIA 5 μM rats, MVIIA 10 μM rats, and MVIIA 20 μM rats).

To investigate the best time for MVIIA application, 24 rats were randomly distributed into four groups: SHAM rats and rats subjected to SCI and injected with placebo (PLA rats) or MVIIA 10 μM 1 h or 4 h after SCI (MVIIA 10 μM 1h rats and MVIIA 10 μM 4h rats, respectively) (Fig 2).

### MVIIA side effect assessment

The intrathecal MVIIA injection side effects were evaluated during the adaptation period, on the day before the surgery, during 5 h after the toxin was applied, and every day until euthanasia was performed. The effects were assessed by open field observation for 15 min. Generalized shaking body behavior, coordination problems, and tail movement were noted and scored as absent, discrete, moderate, or severe [43].

### Locomotor activity assessment

The rats were individually transferred to the viewing arena (open field with 100-cm diameter) on the day before the surgery and every 24 h, until euthanasia was performed. The Basso, Beattie, and Bresnahan (BBB) score was employed [44]. During the open field test, the rats were encouraged to locomote continuously as recommended by Basso et al. [44]. The rats that remained stationary for longer than 15-20 s were enticed to move by having them follow a pencil. If the rat failed to respond, it was picked up and placed in the center of the open field. The observations were recorded for 4 min and were evaluated by two examiners. Toe clearance, paw position, and forelimb-hind limb coordination were assessed with the aid of the BBB scale, which spanned from zero to 21 (S1 Table).

### Cerebrospinal fluid and spinal fragment collection

The rats received xylazine (8 mg/kg, intraperitoneal injection) and were euthanized by administration of a thiopental sodium overdose (100 mg/kg, intraperitoneal injection) 48 h after SCI. After that, the rat head was flexed downward at approximately 45°, to reveal a palpable depressible surface with the appearance of a rhomb between the occipital protuberances and the spine of the atlas. The 22G needle was inserted in the cisterna magna for cerebrospinal fluid (CSF) collection and connected to a 1-mL syringe. Then, 50-80 μL of non-contaminated sample was drawn into the syringe by simple and careful aspiration.

**Fig 3:**
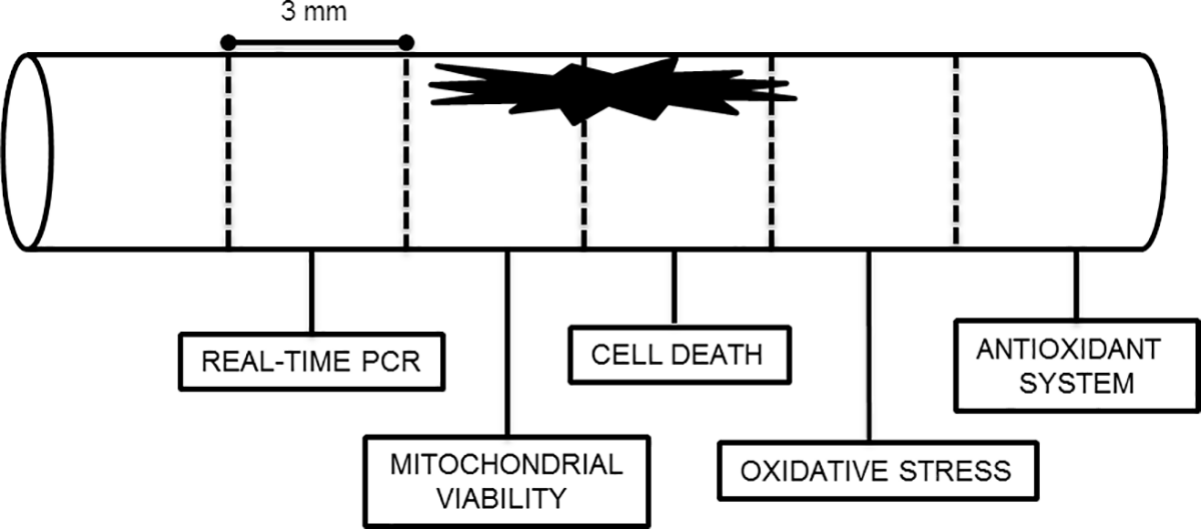
Design of the spinal segments use for each type of test. From cranial to caudal: real-time PCR, mitochondrial viability, cell death, oxidative stress and antioxidant system.

Immediately, about 6 mm of the lesion epicenter in the spinal cord fragment was collected and divided into cranial fragment, to assess mitochondrial viability, and caudal fragment, to evaluate cell death. To optimize the number of animals used, the segment adjacent to the cranial epicenter (3mm) was used for real-time PCR and the adjacent to the oxidative stresse (ROS and lipid peroxidation) evaluation (3mm) and the next caudal segment for the of the antioxidant system (3mm) (Fig 3).

### Glutamate measurement

Glutamate was enzymatically measured in the CSF by monitoring the fluorescence increase due to NADPH^+^ production in the presence of glutamate dehydrogenase and NADP^+^ on a spectrofluorimeter (Shimadzu RF-5301PC, Japan). For this assay, 1.8 mL of Krebs Ringer HEPES (KRH) incubation solution without calcium (NaCl 124 mM, KCl 4 mM, MgSO_4_ 1.2 mM, and HEPES 10 mM), 5 μL of NADP (1 mM), 22 μL of glutamate dehydrogenase, 5 μL of the CSF sample, and 5 μL of standard glutamate were added to an optical cell. The excitation and emission wavelengths were set at 360 and 450 nm, respectively. The glutamate level was calculated as pmol of glutamate per milligram of protein.

DOI; https://dx.doi.org/10.17504/protocols.io.rufd6tn

### Cell viability assessment

About 6 mm of the lesion epicenter in the spinal cord fragment was collected and divided into cranial fragment, to assxess mitochondrial viability, and caudal fragment, to evaluate cell death.

#### Mitochondrial viability

Mitochondrial viability was determined through 2,3,5-triphenyltetrazolium chloride (TTC) conversion to insoluble formazan. TTC reduction depends on the mitochondrial respiratory activity and is proportional to the number of viable cells. After euthanasia, the spinal cord fragment collected from the lesion epicenter was subjected to an artificial cerebrospinal fluid (ACSF) and sliced (400-μM thick) with the aid of a tissue cutter. After processing in the perfusion chamber, the slices were incubated in a TTC 2% solution at 37°C for 90 min. TTC was removed after this period, and the tissue was washed with 0.9% saline solution, added with 1.5 mL of a 1:1 dimethylsulfoxide (DMSO)/ethanol solution, and incubated at room temperature and in the dark for 48 h, to solubilize formazan. The absorbance was read in a spectrophotometer at 485 nm, and the results were normalized by tissue weight.

DOI; https://dx.doi.org/10.17504/protocols.io.ruud6ww

#### Cell death

To analyze cell death, 400-μm slices were stained with 3 μL of ethidium homodimer-1 (6 μM; live/dead assay, Molecular Probes, Eugene, OR) diluted in 1 mL of carbogenic mixture for 30 min and washed with 2 mL of 95% O_2_/5% CO_2_ ACSF containing glucose 10 mM at room temperature for 15 min. During the staining procedure, the slices were protected from light.

To quantify the dead cells, their nuclei were identified by fluorescence staining with ethidium homodimer. The images were acquired with a Zeiss Axiovert 200 M Microscope and the Apotome system, which afforded consecutive 10-μm thick optical sections of a Z-series. These sections were analyzed at the ethidium homodimer excitation/emission wavelength (568/598 nm), with 20x dry objectives. The Image J software was used to combine consecutive optical sections from a Z-series and to create image constructions. All the nuclei in this whole field were counted. The morphological analysis of the dead cells was performed in two fields of the lateral funiculus in gray matter.

DOI; https://dx.doi.org/10.17504/protocols.io.ruhd6t6

### Free radical content, lipid peroxidation, and antioxidant enzyme activity measurement

#### Reactive oxygen species assessment

A caudal adjacent spinal cord to epicenter fragment was collected from the lesion epicenter, and dichlorofluorescein diacetate (DCF-DA) levels were determined as an indicator of peroxide production by cell components [45]. The fragment was immediately cooled to -20°C in TRIS-HCl buffer solution (10 mM, pH 7.4). After homogenization, the fragment was subjected to centrifugation (10,000 rpm, 5 ^o^C, 10 min), and a 20-μL aliquot of this supernatant was added to 80 μL of DCF-DA 125 μM. Triplicate plates were incubated in the dark at 37 ^o^C for 1 h until the fluorescence (488/525 nm) was measured in a Victor X4 apparatus (Perkin-Elmer). The results were normalized by protein content.

DOI; https://dx.doi.org/10.17504/protocols.io.ruid6ue

#### Lipid peroxidation assessment

Lipid peroxidation (LP) was determined by quantifying the malondialdehyde (MDA) content in the homogenate supernatant in the caudal adjacent segment to epicenter by colorimetric reaction with thiobarbituric acid (TBA) at high temperatures. After storage at -20 ^o^C, the sample was homogenized in a sonicator (Branson Sonifier Model 250; three 4-s cycles), and the homogenate was centrifuged at 2500 rpm and 4 ^o^C for 10 min. Initially, 134 μL of TBA 0.8%, 134 μL of acetic acid buffer (125 mL of H_2_O, 18.84 mL of glacial acetic acid, and 5.63 mL of HCl PA 12 N; pH 3.4), and 54 μL of H_2_O were pipetted. After incubation, 54 μL of the sample and 54 μL of sodium dodecyl sulfate (SDS) 8.1% were added, and the solution was left at 95 ^o^C for 90 min. The MDA content was measured at a wavelength of 532 nm, as described by Ohkawa et al. [46]. The results were normalized by protein content.

DOI; https://dx.doi.org/10.17504/protocols.io.rujd6un

### Antioxidant enzyme activities

Catalase activity was assayed as described by Aebi [47]. Briefly, the reaction started upon addition of the supernatant (3 μL) to 20 μL of H_2_O_2_ 10 mM prepared in 600 μL of potassium phosphate buffer (50 mM; pH 7.0) with Tween 0.002% and EDTA 1%. The H_2_O_2_ decomposition rate was measured by spectrophotometry, and the optical density at 240 nm was recorded within 15-s intervals over 150 s. The results were normalized by protein content and expressed as a percentage of the placebo group.

DOI; https://dx.doi.org/10.17504/protocols.io.rukd6uw

Superoxide dismutase (SOD) activity was evaluated by means of a previously described spectrophotometric method [48]. The spinal cord segment homogenate was incubated in a solution containing potassium phosphate buffer 100 mM and EDTA 50 mM, pH 7.4. The reaction was initiated by addition of pyrogallol 2 mM. Pyrogallol oxidation was measured at 420 nm (UV/visible U-200L Spectrophotometer, Hitachinaka, Japan) for 5 min, at 30-s intervals. A 50% inhibition was defined as one unit (U) of SOD, and the results were normalized by protein content and expressed as a percentage of the placebo group.

DOI; https://dx.doi.org/10.17504/protocols.io.rund6ve

Glutathione peroxidase (GPX) activity was determined according to Paglia and Valentine [49]. Briefly, a reaction solution was prepared in 20 mL of phosphate buffer (100 mM; pH 7.0) containing EDTA 5 mM, 3.12 g of NADPH, 10 μL of glutathione reductase (500 U/mg of protein/mL), 250 μL of NaN_3_ 100 mM, and 7.68 g of reduced glutathione. Then, 510 μL of the reaction solution, 30 μL of milli-Q water, and 30 μL of homogenate were added to the optical cell. The enzymatic reaction was initiated with the addition of 60 μL of H_2_O_2_ 4 mM. NADPH conversion to NADP was measured in a spectrophotometer (Hitachi, model U-2001, Hitachinaka City, Japan) for 5 min. The enzyme unit was determined as the oxidation of 1 mol of NADPH per minute and was calculated on the basis of the NADPH absorbance at 340 nm. The results were normalized by the protein content in the sample and expressed as a percentage of the placebo group.

DOI; https://dx.doi.org/10.17504/protocols.io.ruqd6vw

Glutathione reductase (GR) activity was investigated according to Aebi [47]. Briefly, a reaction solution was prepared with 2.13 mg of NADPH in 20 mL of potassium phosphate buffer 150 mM (pH 7.0) and EDTA 1.5 mM. A 50-μL aliquot of the sample was added in a working reagent containing NAPDH 0.15 mM, potassium phosphate buffer 0.15 M (pH 7.0), and 100 μL of water. Kinetics was analyzed in a spectrophotometer at 340 nm, for 120 s, at 30-s intervals. Then, oxidized glutathione was added, and analysis was allowed to continue for 120 s. The enzyme unit was determined as the oxidation of 1 mol of NADPH per minute and was calculated on the basis of the molar absorptivity of NADPH at 340 nm. The results were normalized by the protein content in the sample and expressed as a percentage of the placebo group.

DOI; https://dx.doi.org/10.17504/protocols.io.rurd6v6

Glutathione S-transferase (GST) activity was examined according to Habig et al. [50]). Briefly, 30 μL of the sample was added to 240 μL of potassium phosphate buffer solution (100 mL of monobasic potassium phosphate buffer, 100 mL of dibasic phosphate buffer; pH 7.5) and 318 μL of milli-Q water. After homogenization, 6 μL of GSH 100 mM and 6 μL of 1-chloro-2,4-dinitrobenzene (CDNB) 100 mM were added. The reagents were directly placed into the optical cells, and the absorbance was read in a spectrophotometer (Hitachi, model U-2001, Hitachinaka City, Japan) at 340 nm, for 120 s, at 30-s intervals. The enzymatic activity was determined according to the CDNB extinction coefficient.

DOI; https://dx.doi.org/10.17504/protocols.io.rusd6we

#### Protein assessment by the Bradford method

Protein was determined by the colorimetric method described by Bradford [51]. To this end, 2 μL of supernatant, obtained by suspending the pellet from the caudal segment of the spinal cord, was used. Triplicate samples were placed in plates by addition of 500 μL of NaCl 0.15 M and 500 μL of Bradford reagent to each sample. The mixture was incubated for 5 min and stirred, and the absorbance was read in a spectrophotometer at a wavelength of 595 nm. The protein concentration was calculated by using a standard curve dilution of 1 mg of BSA/mL with 1, 3, 5, 7, and 10 μg.

DOI; https://dx.doi.org/10.17504/protocols.io.rutd6wn

### Bax, Bcl-xl, caspase-3, caspase-8, caspase-9, caspase-12, and nNOS expressions by real-time PCR

To determine the best MVIIA administration dose, and application time, a cranial segment of the lesion epicenter was collected (3 mm) from the six rats in each group (SHAM rats, PLA rats, MVIIA 10 μM 1h rats, and MVIIA 10 μM 4h rats) to evaluate the gene expressions of Bax, Bcl-xl, caspase-3, caspase-8, caspase-9, caspase-12, and nNOS (Table 1), which are all related to apoptosis.

**Table 1:** Primer sequences for real time PCR analysis.

Gene	Primer sequence (5’ - 3’)	Access number
**Bax**	F: CCAAGAAGCTGAGCGAGTGTCTC	NM_017059.1
	R: AGTTGCCATCAGCAAACATGTCA	
**Bcl-xl**	F: CCCCAGAAGAAACTGAACCA	NM_001033670.1
	R: AGTTTACCCCATCCCGAAAG	
**Caspase-3**	F: TGGAGGAGGCTGACCGGCAA	NM_012922.2
	R: CTCTGTACCTCGGCAGGCCTGAAT	
**Caspase-8**	F: TAAGACCTTTAAGGAGCTTCATTTTGA	NM_022277.1
	R: AGGATACTAGAACCTCATGGATTTGAC	
**Caspase-9**	F: TGGAGGAGGCTGACCCGGCAA	NM_031632.1
	R: CCACAGCTCCGCGACTTGCA	
**Caspase-12**	F: AGGGATAGCCACTGCTGATACAGA	NM_130422.1
	R: CTGTCTCCACATGGGCCTTTGTT	
**nNOS**	F: TCCCTCTAGCCAAAGAATTTCTCG	NM_052799
	R: GGTAGGTGCTGGTGCTTTCAA	
**Beta-actin**	F: GCGTCCACCCGCGAGTACAA	NM_031144.2
	R: ACATGCCGGAGCCGTTGTCG	

Total RNA was extracted with TRIzol reagent (Invitrogen Corporation, Carlsbad, CA, USA), chloroform, and isopropanol. The precipitate was washed with ethanol, air-dried, and re-diluted in diethyplyrocarbonate (DEPC)-treated distilled water. The amount and purity of extracted RNA was quantified by spectrophotometry (GeneQuantTM pro RNA/DNA; GE Healthcare, Piscataway, NJ, USA). RNA reverse transcription and real-time PCR reactions were performed with the aid of the Two-Step qRT-PCR Kit with SYBR Green (Invitrögen, Carlsbad, CA, USA). Table 1 summarizes the primer sequences. For real-time PCR, the data were analyzed with 7500 software v.2.0.1 Applied Biosystems; the comparative Cycle threshold (Ct) method was employed. The mRNA level is presented as the number of copies per 103 copies of β-actin mRNA by considering n = 3.3 Ct and 10n = difference in the number of mRNA copies.

### Statistical analysis

The data were tested for normal distribution by the Kolmogorov-Smirnov test; significance was tested with either the Student-Newman-Keuls (SNK) test in the case of normal distribution for three groups or more or unpaired t test for two experimental groups. As for non-normal distribution, the Mann-Whitney test was used for post hoc analysis. To evaluate the locomotor activity score, the Kruskall-Wallis test followed by Dunn’s test was carried out. The statistical analyses were conducted with the Prism software (GraphPad Software, Inc., San Diego, CA, USA); the 95% confidence level was considered significant.

## Results

### First stage: Determining the best MVIIA dose

#### MVIIA side effects

We used open field observation to evaluate the MVIIA side effects and to verify different clinical signs. Side effects were not evident in MVIIA 10 μM rats. However, MVIIA 20 μM rats presented moderate (33.33%) to severe (66.67%) signs like generalized shaking body behavior, coordination problems, and tail movement, which started between 40 and 90 min after MVIIA injection, presenting the moderate to severe signs until 4 h after application, and gradually decreasing for 24 h (S2 Table). No animal showed signs of exacerbated stress, pain, incompatible with animal welfare.

#### Locomotor activity

**Fig 4:**
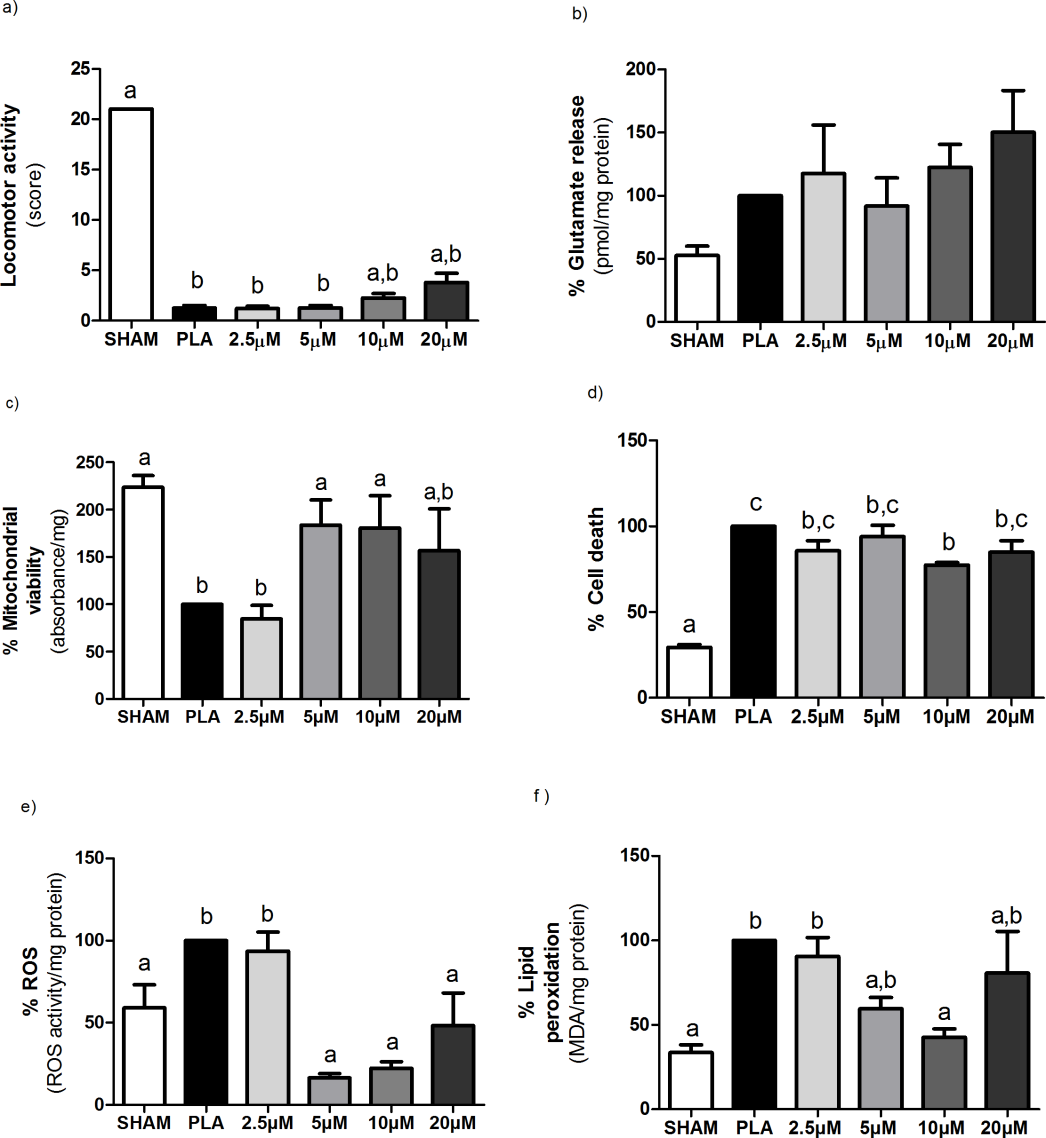
Different MVIIA doses effect on locomotor activity, glutamate release, mitochondrial viability, cell death, reactive oxygen species (ROS), and lipid peroxidation after spinal cord injury in Wistar rats. Graphic representation of the results obtained for the rats subjected to dorsal laminectomy (SHAM rats, negative control) or to spinal cord injury (SCI) and subsequent injection of PBS (placebo, PLA rats) or MVIIA (MVIIA 2.5, 5, 10, and 20 μM rats) 4 h after the trauma. a) Plot of the BBB scale score (mean ± standard deviation) of deambulation in open field 24 h after surgery. The trauma groups did not differ significantly (Kruskal-Walis test, p < 0.01; SHAM, PLA, 2.5, 5, 10, and 20 μM MVIIA: 21 ± 0, 1.22 ± 0.67, 1.22 ± 0.67, 1.25 ± 0.5, 2.25 ± 1.28, and 3.8 ± 2.05, respectively). b) The glutamate concentration in the MVIIA groups was practically the same 48 h after SCI (Student-Newman-Keuls test, p > 0.05). c) Quantification of mitochondrial viability 48 h after surgery shows cell preservation in SHAM, 5 and 10 μM MVIIA in relation to PLA (100) (Student-Newman-Keuls test; PLA vs SHAM, 100% vs 223.61% ± 28.24, p < 0.01; PLA vs 5 μM MVIIA, 100% vs 183.86% ± 59.13, p < 0.05; PLA vs 10 μM MVIIA, 100% vs 180.70% ± 68.20, p < 0.05). d) The analysis of cell death 48 h after the trauma revealed significant reduction in SHAM and 10 μM MVIIA in relation to PLA (Student-Newman-Keuls test; PLA vs SHAM, 100% vs 29.46% ± 2.99, p < 0.01; PLA vs 10 μM MVIIA, 100% vs 77.43% ± 3.62, p < 0.05). e) ROS formation in SHAM, 5, 10, and 20 μM MVIIA differed from PLA rats (Student-Newman-Keuls test; PLA vs SHAM, 100% vs 59.05% ± 27.9, p < 0.05; PLA vs 5 μM MVIIA, 100% vs 16.43% ± 5.75, p < 0.01; PLA vs 10 μM MVIIA, 100% vs 22.34% ± 9.8, p < 0.01; PLA vs 20 μM MVIIA, 100% vs 16.43% ± 5.75, p < 0.01). f) The analysis of lipid peroxidation 48 h after the trauma revealed significant reduction in SHAM and 10 μM MVIIA in relation to PLA (Student-Newman-Keuls test; PLA vs SHAM, 100% vs 33.59% ± 9.14, p < 0.01; PLA vs 10 μM MVIIA, 100% vs 42.69% ± 12.38, p < 0.01). The data were normalized in relation to PLA (100). Different lowercases express statistical difference.

Before SCI, all the rats displayed normal neurological parameters, represented by score 21 in the BBB scale [44]. The BBB scores of PLA rats, MVIIA 2.5 μM rats, MVIIA 5 μM rats, MVIIA 10 μM rats, and MVIIA 20 μM rats did not differ significantly (1.22 ± 0.67, 1.22 ± 0.67, 1.25 ± 0.5, 2.25 ± 1.28, and 3.8 ± 2.05, respectively) 24 h after SCI, which attested to lesion standardization for treatment assessment (Fig 4A). Slight movement of one or two articulations (score 1) or wide movement of one articulation (score 2) corresponded to moderate to severe injury. SHAM rats scored maximum BBB after the surgical procedure, which indicated that laminectomy did not injure the spinal tissue.

#### MVIIA effects on glutamate release

Compared to PLA rats (100%), the mean glutamate concentration values (± standard deviation) in the CSF of SHAM rats, MVIIA 2.5 μM rats, MVIIA 5 μM rats, MVIIA 10 μM rats, and MVIIA 20 μM rats were 52.6% ± 14.81, 117.61% ± 76.88, 91.92% ± 49.71, 122.5% ± 44.11, and 150.30% ± 66.1, respectively (Fig 4B). The glutamate concentration in the MVIIA groups was practically the same 48 h after SCI.

#### Mitochondrial viability

Compared to PLA rats (100%), cell preservation was 1.8 times higher in MVIIA 5 μM rats and MVIIA 10 μM rats (183.86% ± 59.13 and 180.70% ± 68.20, respectively; p < 0.05). For the MVIIA 2.5 μM rats and MVIIA 20 μM rats, the mean cell preservation values (± standard deviation) were 84.97% ± 41.50 and 157.08% ± 87.87, respectively. The mean cell preservation of MVIIA 2.5 μM rats differed not only from the mean cell preservation of PLA rats (p < 0.01), but also from the mean cell preservation of SHAM rats (223.61% ± 28.24; p < 0.01) (Fig 4C).

#### Cell death

Comparison of the mean cell death values revealed significant differences (p < 0.01) between SHAM rats (29.46% ± 2.99) and rats subjected to SCI. MVIIA 10 μM rats had lower cell death values than PLA rats (22.57% ± 3.62 and 100%, respectively; p < 0.05). Cell death values (mean ± standard deviation) were 85.81% ± 15.62, 93.96% ± 14.99, 77.43% ± 3.62, and 84.97% ± 13.47 for MVIIA 2.5 μM rats, MVIIA 5 μM rats, MVIIA 10 μM rats, and MVIIA 20 μM rats, respectively (Fig 4D) (S1 Fig).

#### MVIIA effect on reactive oxygen species after spinal cord injury

ROS formation in MVIIA 5 μM rats (16.43% ± 5.75; p < 0.01), MVIIA 10 μM rats (22.34% ± 9.8; p < 0.01), and MVIIA 20 μM rats (48.29% ± 39.58; p < 0.05) reduced to the levels detected in SHAM rats (59.05% ± 27.9). ROS formation in all the groups above differed from ROS formation in PLA rats (100%) and MVIIA 2.5 μM rats (93.59% ± 34.96) (Fig 4E).

#### MVIIA effect on lipid peroxidation after spinal cord injury

Fig 4F shows that LP decreased 2.34 times in MVIIA 10 μM rats as compared to PLA rats (42.69% ± 12.38 and 100%, respectively; p < 0.01). In turn, LP values were similar in MVIIA 10 μM rats and SHAM rats. This assay afforded the following results (mean ± standard deviation): 33.59% ± 9.14, 90.54% ± 33.93, 59.52% ± 14.97, and 80.72% ± 49.42 for SHAM rats, MVIIA 2.5 μM rats, MVIIA 5 μM rats, and MVIIA 20 μM rats, respectively.

### Second stage: Determining the best application time

The first experiment demonstrated that MVIIA 10 μM had a neuroprotective effect after SCI. Therefore, we selected this MVIIA dose, intrathecal route, to compare the application times 1 h and 4 h after SCI.

#### MVIIA application and side effects

Rats injected with intrathecal MVIIA 10 μM 4 h after SCI (MVIIA 10 μM 4h rats) did not present any side effects. However, in the case of intrathecal MVIIA 10 μM 1 h after SCI (MVIIA 10 μM 1h rats), one rat (16.7%) exhibited moderate tail tremors that were reverted within 24 h.

#### Spinal cord injury complications – urinary retention

Among the rats subjected to SCI, 100%, 66.66%, and 42.86% of the PLA rats, MVIIA 10 μM 1h rats, and MVIIA 10 μM 4h rats presented with urinary retention, respectively. In the two former groups, urinary retention was associated with hematuria (60% and 33.33%, respectively).

#### Locomotor activity assessment

**Fig 5:**
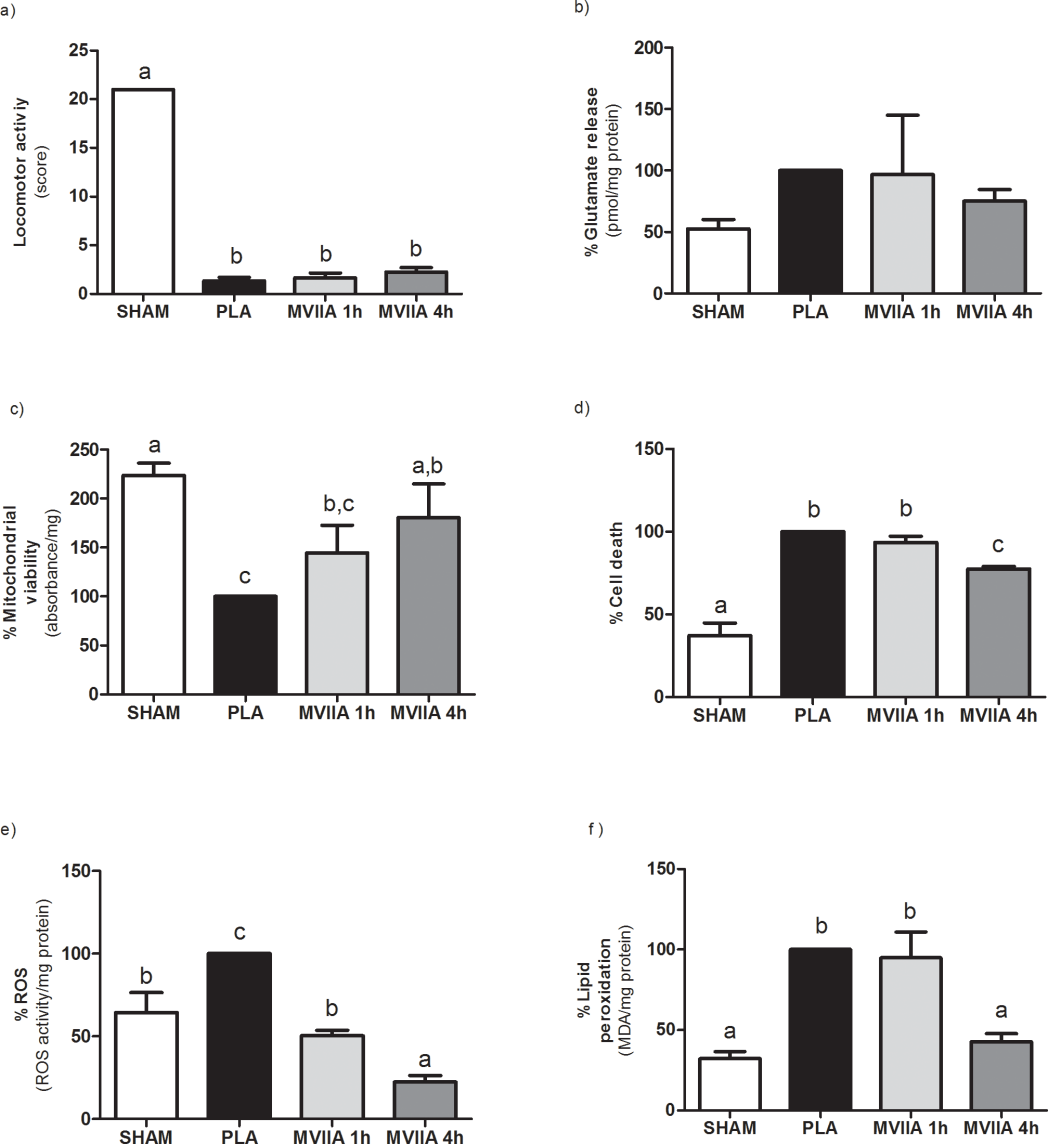
MVIIA effect on locomotor activity, glutamate release, mitochondrial viability, cell death, reactive oxygen species (ROS) and lipid peroxidation after spinal cord injury in Wistar rats treated with MVIIA at different times. Graphic representation of the results obtained for the rats subjected to dorsal laminectomy (SHAM rats, negative control) or to SCI and subsequent injection of PBS (placebo, PLA rats) or MVIIA 10 μM 1 h or 4 h after surgery (MVIIA 10 μM 1h rats or MVIIA 10 μM 4h rats). a) Plot of the BBB scale score (mean ± standard deviation) of deambulation in open field 24 h after surgery. The trauma groups did not differ significantly (Kruskal-Walis test, p < 0.001; SHAM, PLA, 10 μM 1h or 10 μM 4h MVIIA: 21 ± 0, 1.33 ± 0.82, 1.67 ± 1.21, 2 ± 2.5, respectively). b) The glutamate concentration in the MVIIA groups was practically the same 48 h after SCI (Student-Newman-Keuls test, p > 0.05). c) Quantification of mitochondrial viability 48 h after surgery shows cell preservation in SHAM, 10 μM 4h MVIIA in relation to PLA (100) (Student-Newman-Keuls test; PLA vs SHAM, 100% vs 223.61% ± 28.24, p < 0.01; PLA vs 10 μM 4h MVIIA, 100% vs 180.70% ± 68.20, p < 0.05). d) The analysis of cell death 48 h after the trauma revealed significant reduction in SHAM and 10 μM 4h MVIIA in relation to PLA (Student-Newman-Keuls test; PLA vs SHAM, 100% vs 37.01% ± 15.31, p < 0.01; PLA vs 10 μM 4h MVIIA, 100% vs 77.43% ± 3.62, p < 0.05). e) ROS formation in SHAM, 10 μM 1h or 10 μM 4h MVIIA differed from PLA rats (Student-Newman-Keuls test; PLA vs SHAM, 100% vs 64.34% ± 26.9, p < 0.001; PLA vs 10 μM 1h MVIIA, 100% vs 50.32% ± 7.36, p < 0.001; PLA vs 10 μM 4h MVIIA, 100% vs 22.34% ± 9.8, p < 0.001). f) The analysis of lipid peroxidation 48 h after the trauma revealed significant reduction in SHAM and 10 μM MVIIA in relation to PLA (Student-Newman-Keuls test; PLA vs SHAM, 100% vs 33.59% ± 9.14, p < 0.01; PLA vs 10 μM MVIIA, 100% vs 42.69% ± 12.38, p < 0.01). The data were normalized in relation to PLA (100). Different lowercases express statistical difference.

Before SCI, all the rats presented normal neurological parameters and scored 21 in the BBB scale. SCI standardization occurred 24 h after the surgery, as seen from the similar BBB scores of PLA rats (1.33 ± 0.82), MVIIA 10 μM 1h rats (1.67 ± 1.21), and MVIIA 10 μM 4h rats (2 ± 2.5) (Fig 5A). Slight movement of one or two articulations (score 1) or wide movement of one articulation (score 2) corresponded to moderate to severe injury. SHAM rats scored maximum BBB after the surgical procedure, which indicated that laminectomy did not injure the spinal tissue.

#### MVIIA effect on glutamate release after spinal cord injury

Glutamate levels did not decrease significantly 48 h after SCI. In relation to PLA rats (100%), the mean glutamate concentration (± standard deviation) in the CSF of SHAM rats, MVIIA 10 μM 1h rats, and MVIIA 10 μM 4h rats were 52.6% ± 14.81, 96.75% ± 96.55, and 75.24% ± 24.89, respectively (Fig 5B).

#### Mitochondrial viability

MVIIA 10 μM 4h rats had significantly higher (1.8 times) cell preservation (180.70% ± 68.20) as compared to PLA rats (100%) (Fig 5C). As for MVIIA 10 μM 1h, cell preservation was only statistically different as compared to SHAM rats (144.63% ± 56.30 and 223.61% ± 28.24, respectively; p < 0.05).

#### Cell death

Comparison of the mean cell death values revealed a significant reduction between SHAM rats (37.01% ± 15.31) and MVIIA 10 μM 4h (77.43% ± 3.62) rats compared to other rats subjected to SCI (p < 0.01). Considering the MVIIA injection times, MVIIA 10 μM 1h rats had the same cell death (97.42% ± 12.09, respectively; p < 0.01) as compared to PLA rats (100%) (Fig 5D) (S2 Fig).

#### MVIIA effect on reactive oxygen species after spinal cord injury

MVIIA 10 μM 1h rats and MVIIA 10 μM 4h rats had reduced ROS production (50.32% ± 7.36 and 22.43% ± 9.8, respectively; p < 0.001) as compared to PLA rats (100%). Nevertheless, MVIIA 10 μM application 4 h after SCI was even more effective: ROS values were significantly lower in MVIIA 10 μM 4h rats as compared to SHAM rats (64.34% ± 26.9; p < 0.001) (Fig 5E).

#### MVIIA effect on lipid peroxidation after spinal cord injury

MVIIA 10 μM injection 4 h after SCI diminished LP 2.34 times as compared to placebo (42.69% ± 12.38 and 100%, respectively; p < 0.001), whereas MVIIA 10 μM injection only 1 h after SCI did not elicit statistically different results (94.95% ± 35.75) (Fig 5F).

#### MVIIA effect on the antioxidant system after spinal cord injury

**Fig 6:**
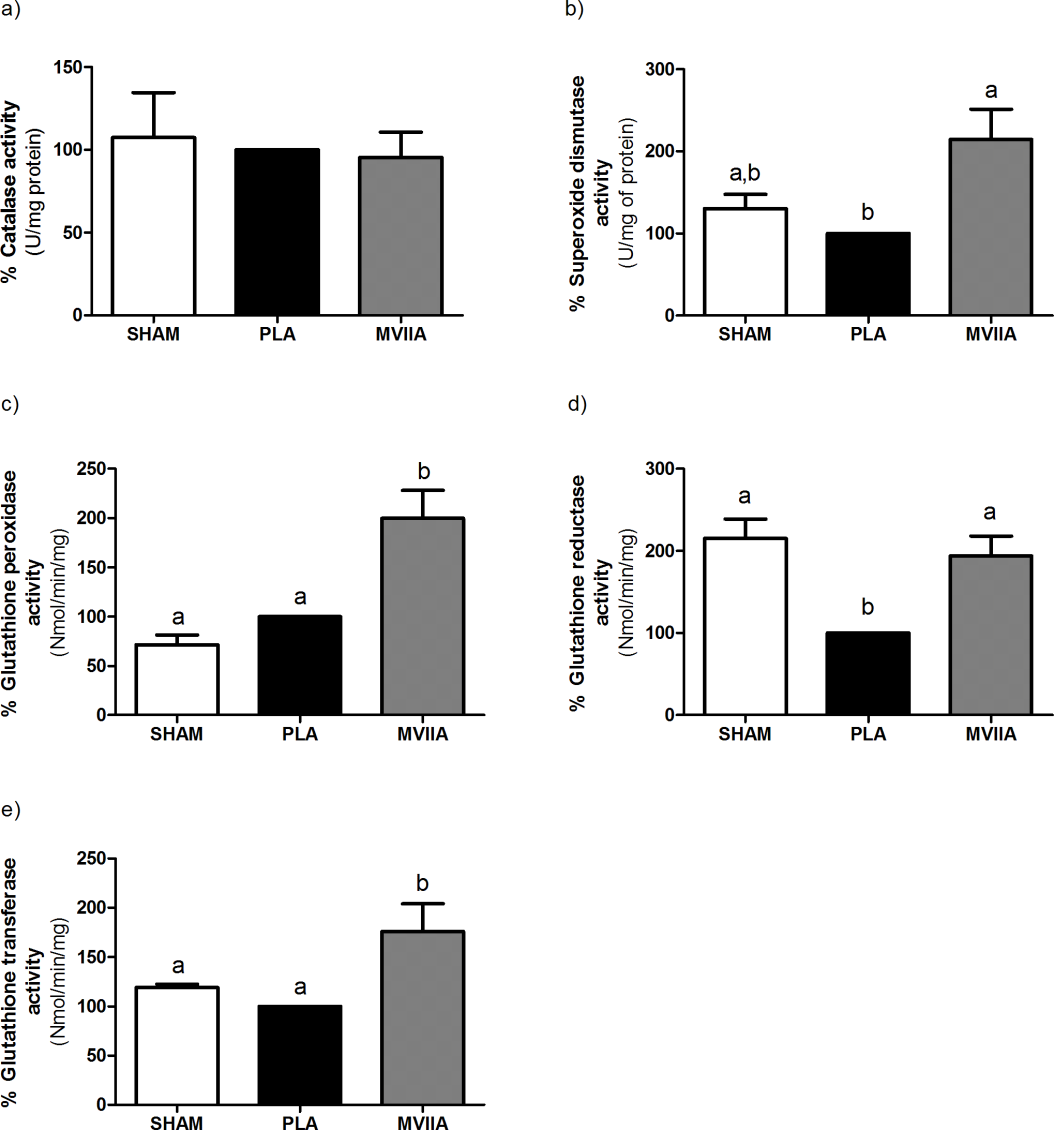
MVIIA effect on superoxide dismutase, catalase, glutathione peroxidase, glutathione reductase, and glutathione transferase activities 48 h after spinal cord injury. Graphic representation of the results obtained for the rats subjected to dorsal laminectomy (SHAM rats, negative control) or to spinal cord injury and subsequent injection of PBS (placebo, PLA rats) or MVIIA 10 μM (MVIIA 10 μM rats). a) There were no catalase activity statistical differences among the groups PLA, MVIIA, and SHAM rats 48 h after SCI (Student-Newman-Keuls test, p > 0.05). b) Quantification of superoxide dismustase activity 48 h after surgery shows higher levels in MVIIA rats in relation to PLA (100) (Student-Newman-Keuls test; PLA vs MVIIA 100% vs 188.41% ± 72.05, p < 0.05). c) The analysis of glutathione peroxidase activity 48 h after the trauma revealed significant increase in MVIIA group when compared to PLA (Student-Newman-Keuls test; PLA vs MVIIA, 100% vs 199.96% ± 68.65, p < 0.01). d) Glutathione reductase activity was significantly lower in PLA rats as compared to SHAM rats (PLA vs SHAM, 100% and 215.01% ± 58.54, p < 0.01) and to MVIIA (PLA vs MVIIA, 100% vs 193.86% ± 59.39, p< 0.01, Student-Newman-Keuls test). e) Glutathione transferase activity was significantly higher in SHAM rats and MVIIA in relation to PLA (PLA vs SHAM, 100% vs 119.12% ± 8.46, p< 0.05; PLA vs MVIIA, 100% vs 175.93% vs 68.92%, p < 0.05, Student-Newman-Keuls test). The data are normalized in relation to PLA (100). Different lowercases express statistical difference.

There were no statistical differences among the PLA rats, MVIIA rats, and SHAM rats in terms of mean CAT activity (± standard deviation). The values were 100%, 95.42% ± 37.61, and 107.40% ± 60.64, respectively (p > 0.05) (Fig 6A).

Rats treated with MVIIA 10 μM had 1.8 higher SOD activity than PLA rats (188.41% ± 72.05 and 100%, respectively; p < 0.05). Therefore, MVIIA increased the activity of the first enzyme of the detoxification pathway. In turn, MVIIA-treated rats had similar SOD activity to SHAM rats (143.07 ± 56.02) (Fig 6B).

On the basis of Fig 6C, MVIIA increased GPX acitivity 1.9 times as compared to placebo (199.96% ± 68.65 and 100%, respectively; p < 0.01).

GR activity was significantly lower in PLA rats as compared to SHAM rats (100% and 215.01% ± 58.54, respectively). These results reinforced the finding that MVIIA elevated GR activity 1.9 times as compared to placebo (193.86% ± 59.39 and 100%, respectively; p = 0.002) (Fig 6D). MVIIA 10 μM rats had 1.7 higher GT activity as compared to PLA rats and higher GT activity as compared to SHAM rats (175.93% ± 68.92, 100%, and 119.12% ± 8.46, respectively; p = 0.013) (Fig 6E).

#### MVIIA effect on the gene expressions of apoptosis-related factors

In the two previous stages, we found that intrathecal MVIIA 10 μM application 4 h after SCI had a neuroprotective effect. Therefore, we investigated the effect of applying intrathecal MVIIA 10 μM 4 h after SCI on the relative gene expressions of apoptosis-related factors such as Bax, Bcl-xl, caspase-3, caspase-8, caspase-9, caspase-12, and nNOS.

**Fig 7:**
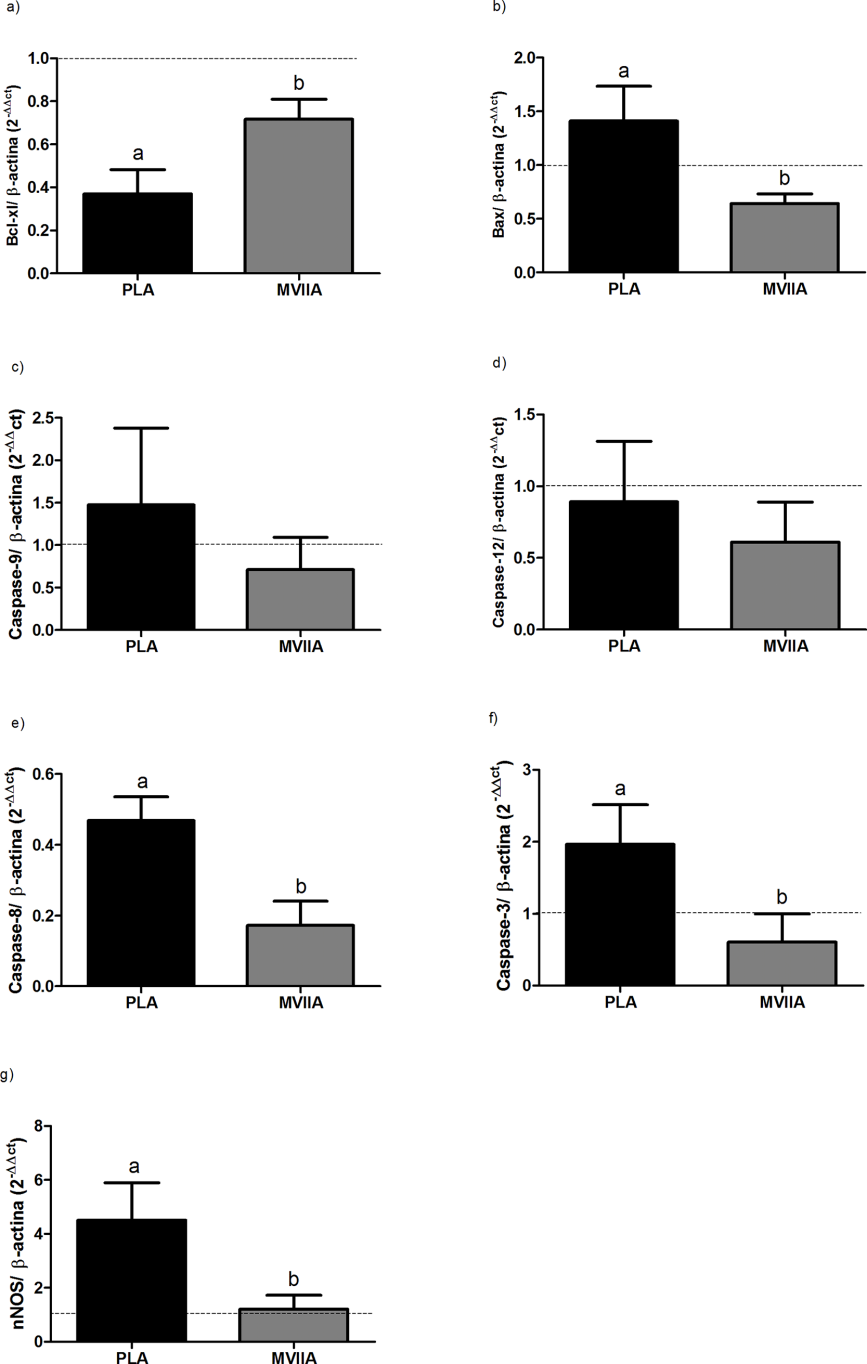
MVIIA effect on the relative gene expressions of Bcl-xl, Bax, caspase-9, caspase-12, caspase-8, caspase-3, and nNOS 48 h after spinal cord injury. Mean relative gene expressions of Bcl-xl (a), Bax (b), caspase-9 (c), caspase-12 (d), caspase-8 (e), caspase-3 (f), and nNOS (g) (± standard deviation) in rats subjected to spinal cord injury associated with intrathecal administration of PBS (placebo, PLA rats) and MVIIA 10μM 4 h after SCI. Lowercases express statistical difference (a: PLA vs MVIIA, 0.72 ± 0.21 vs 0.37 ± 0.25, non-paired *t* test, p < 0.05; b: PLA vs MVIIA, 0.64 ± 0.18 vs 1.41 vs 0.64, Mann-Whitney test, p < 0.05; c: PLA vs MVIIA, 1.47 ± 2.21 vs 0.71 ± 0.76, Mann-Whitney test, p > 0.05; d: PLA vs MVIIA, 0.89 ± 0.94 vs 0.61 ± 0.62, non-paired *t* test, p > 0.05; e: PLA vs MVIIA, 0.47 ± 0.13 vs 0.17 ± 0.13, Mann-Whitney test, p < 0.05; f: PLA vs MVIIA, 1.96 ± 0.55 vs 0.60 ± 0.39, non-paired *t* test, p < 0.05; g: PLA vs MVIIA, 4.51 ± 2.77 vs 1.22 ± 1.14 non-paired *t* test, p < 0.05).

The relative expression of the anti-apoptotic protein Bcl-xl was higher in MVIIA 10 μM rats as compared to PLA rats (0.72 ± 0.21 and 0.37 ± 0.25, respectively; p < 0.05) (Fig 7A). At the same time, the relative expression of Bax, a pro-apoptotic protein, decreased in MVIIA 10 μM rats as compared to PLA rats (0.64 ± 0.18 and 1.41 ± 0.64, respectively; p < 0.05) (Fig 7B).

The caspase-9 relative expression was 1.47 ± 2.21 and 0.71 ± 0.76 in PLA rats and MVIIA 10 μM rats, respectively (Fig 7C), whereas the caspase-12 relative expression was 0.89 ± 0.94 and 0.61 ± 0.62 in PLA rats and MVIIA 10 μM rats, respectively (Fig 7D). In the case of both caspases, differences between PLA rats and MVIIA 10 μM rats were not significant.

The relative caspase-8 expression was lower in MVIIA 10 μM rats as compared to PLA rats (0.17 ± 0.13 and 0.47 ± 0.13, respectively; p < 0.05) (Fig 7E). Similarly, the relative caspase-3 expression was lower in MVIIA 10 μM rats as compared to PLA rats (0.60 ± 0.39 and 1.96 ± 0.55, respectively; p < 0.05) (Fig 7F).

MVIIA 10 μM reduced the relative nNOS expression as compared to placebo (1.22 ± 1.14 and 4.51 ± 2.77, respectively; p < 0.05) (Fig 7G).

## Discussion

In this study, we have demonstrated for the first time and to the best of our knowledge that intrathecal MVIIA administration exerts a neuroprotective effect in the rat spinal cord compression paradigm and is an excellent alternative for acute SCI treatment protocols. Clinical studies on other animals and humans are necessary to establish MVIIA routine clinical use in veterinary and human medicine. MVIIA is a structurally stable, easy to synthesize, and highly specific calcium channel blocker [13]. All these features make MVIIA an especially promising alternative when it comes to reducing ischemic [29, 31–34] and traumatic [24, 27–30] brain injuries and SCI [35]: MVIIA can hinder exacerbated calcium influx and consequently prevent lesion progression [52; 53]. It is known that high N-type voltage-dependent calcium channel (VDCC) concentrations are expressed in the spinal cord dorsal laminae [54; 55]. These channels play a fundamental role in calcium flux regulation [56], whereas MVIIA is a specific and reversible blocker of these channels [57–59].

In a previous study, our group tested intralesional MVIIA application 5 min after SCI, but results were not significant [36]. According to Valentino et al. [31] and Verweij et al. [30], MVIIA injection 15 min before [30] or even 1 h after [31] ischemic and traumatic brain injuries, respectively, does not preserve mitochondria as compared to MVIIA administration 4 h or 6 h after the trauma event [30]. Therefore, we decided to investigate late MVIIA application, namely 1 h and 4 h after SCI. Direct lumbar intrathecal MVIIA injection between L5-L6, as described by Mestre et al. [42], was essential to our experiments—the toxin has peptidic nature and is not easily available after oral administration, so it has to be directly applied at the target organ [60]. The intrathecal administration route allows rapid, innocuous, reliable, and reproducible MVIIA application with no need for anesthesia. The dilution volume (10 μL) suffices to promote safe MVIIA diffusion to the CSF [35, 42, 61].

On the basis of literature antinociceptive studies demonstrating that MVIIA concentrations spanning from 3 to 200 μM effectively block N-type VDCCs [61–63], we conducted a dose-response study in which we employed MVIIA concentrations ranging from 2.5 to 20 μM. The emergence of side effects limited the use of higher MVIIA concentrations [58, 64–68]. In agreement with Hama and Sagen [63], MVIIA 5 μM did not give rise to complications. In contrast to the tremors and tail movements reported by Malmberg and Yaksh [43] and Souza et al. [62], MVIIA 10 μM only elicited alterations in one rat when we applied the toxin 1 h after SCI. This corroborated with the data published by Souza et al. [61], who described that alterations become more intense and more frequent only at higher MVIIA doses (20 and 40 μM). On average, we verified that the initial tremors started 90 min after MVIIA injection and were reverted within 8 h. However, some rats that received MVIIA 20 μM presented slight tail tremors up to 24 h after administration, which agreed with Malmberg and Yaksh [43] and Scott et al. [65]. We also noted that these tremors persisted for more than 24 h in rats that received MVIIA 40 μM. Due to intense and persistent side effects, we opted to remove the latter group of rats from the experiment and to submit them to earlier euthanasia. Even though the clinical use of MVIIA is limited to low doses, picomolar MVIIA doses are enough to elicit N-type calcium channel inhibition [26, 61, 62].

The compression lesion model used here is well established [36, 38–40] and allowed us to reproduce moderate to severe injury efficiently. The model provides urinary retention and mimics conditions that often occur in humans [69]. Urinary retention was less frequent in MVIIA 10 μM 4h rats, which meant that less damage secondary to SCI emerged in this group as SCI also causes bladder functional deficit due to interruption of ascending and/or descending tracts [70].

Glutamate-mediated excitotoxicity, excess intracellular calcium (which leads to ionic modifications and causes cell apoptosis and necrosis through increased cellular enzyme activation), mitochondrial damage, acidosis, and free radical production are among the various events contributing to secondary neuronal death following SCI [4, 5, 69, 71–74]. The MVIIA neuroprotective mechanism has not been fully established, but N-type VDCC blockade inhibits the release of many neurotransmitters like glutamate [18,75], directly inhibits calcium influx [76], preserves mitochondria in traumatic brain injury [28, 30, 77], and prevents neuronal cell degeneration while improving behavioral and cognitive functions [29]. Here, glutamate levels 48 h after SCI were not different in any of the study groups, even though literature studies have shown that MVIIA reduces glutamate concentrations soon after SCI [18, 62]. Indeed, SCI investigations have reported significant decrease in glutamate levels between 3 h [78–80] and 4 h after injury [81; 82], whereas we only assessed glutamate 48 h after SCI, which could explain why we did not observe differences regarding this neurotransmitter. Some authors have also reported that glutamate inhibition is not the only pathway implicated in MVIIA neuroprotection because high MVIIA doses are necessary for this inhibition to occur [31; 83].

The traumatic brain injury model [30, 77] established the MVIIA neuroprotective mechanism and showed improved mitochondrial viability after MVIIA application. In agreement with this model, we observed that MVIIA 5 and 10 μM administration 4 h after SCI enhanced mitochondrial viability, which reached the same level measured in SHAM rats. These findings contrasted with the results achieved for MVIIA 10 μM injection one hour after SCI. Interestingly, Valentino et al. [31] and Verweij et al. [30] also noted that MVIIA supplied 15 min or 1 h afer SCI does not preserve the mitochondria as much as late MVIIA administration (4 h or 6 h) after brain ischemia and injury. The reason for the delayed MVIIA effect remains unknown. Verweij et al. [30] suggested that this toxin should be more effective during critical calcium periods. Because calcium levels peak 8 h after SCI [84], MVIIA injection 4 h after the trauma should have maximum effect between 3 h [61] and 4 h [26] after its application, to coincide with the peak intracellular calcium concentration.

We conducted another cell viability assay with ethidium homodimer staining, which can easily distinguish between damaged and intact cells through DNA staining [85]. Together with the previous results, this assay allowed us to analyze cell viability and mortality during SCI. MVIIA 10 μM administration 4 h after SCI exerted a neuroprotective effect—cell viability and cell death values were 80% and 23%, respectively, in different spinal cord segments. These data were consistent with reports that MVIIA injection until 24 h after the event provides significant protection during ischemic [31, 32] and traumatic brain injuries [29, 30].

Mitochondria also release factors that activate the apoptosis cascade, to increase injury secondary to SCI even further, including oxidative damage, ROS production, synapse disruption, and cell death [8, 86–89]. Moreover, in our assays we observed improved mitochondrial viability after MVIIA administration. Therefore, we investigated oxidative stress, the antioxidant system, and the apoptosis cascade in order to detail the possible neuroprotective mechanisms implicated in the MVIIA action. Although doses of 5 μM, 20 μM, 10 μM 1h and 10 μM 4h reduced the ROS levels, only the delayed MVIIA 10 μM administration inhibited ROS production to a larger extent that could preserve the cells. Among the generated ROS, peroxynitrite decomposition gives highly toxic free radicals that culminate in LP [7, 90], one of the most harmful events following SCI—LP disrupts the cell membrane and produces neurotoxic factors, such as MDA [91], which we quantified here. As the attenuation of the ROS production is directly related to the decrease of LP, MVIIA 10 μM injection 4 h after SCI abated lipid peroxidation in 2.34 times as compared to PLA rats, being able to reduce the damage generated by oxidative stress. This corroborated the previous data and evidenced the MVIIA neuroprotective effect, which resembled the effect reported for other marine peptides [92, 93].

Because free radicals cause oxidative damage, they are part of a crucial mechanism in neurological diseases. Free radicals have been reported as the first biochemical alterations after SCI [94]. Knowing that MVIIA administration reduces free radical oxidative damage, and that tissues protect themselves by means of antioxidant enzymes [86, 95], we investigated how MVIIA acts on this pathway. Treatment with MVIIA markedly increased SOD and GPX activities: 1.8 and 1.9 times as compared to placebo. We did not detect any differences in the case of CAT activity possibly because we evaluated this enzyme only 48 h after SCI. Apart from not being able to neutralize all the ROS, the SOD and GPX enzymes can react with macromolecules, to generate secondary products that require detoxification in order to prevent further intracellular damage and eventual cell death [96]. For all these reasons, we also assessed the second cell defense line. We found that MVIIA was able to re-establish 90% and 70% of GR and GT activities, respectively. It is known that ROS levels in the spine are 48% higher 4 h after SCI. Nevertheless, defense molecules only arise hours after ROS emerge [86]. Here, MVIIA application improved the cell detoxification mechanism, which suggested that this pathway accounts for reduced secondary damage and neuronal death after SCI and acts in both the first and second cell defense line. This supports the hypothesis that MVIIA can function as a neuroprotective agent in SCI treatment: oxidative stress plays a pivotal role in injuries secondary to SCI, and inhibiting these injuries is a potential intervention strategy. Investigations into therapies that enhance antioxidant defenses or diminish pro-oxidant processes have efficiently prevented, improved, or retarded neurological alterations and promoted neuroprotection [86, 90, 97].

We also examined the apoptosis cascade and observed, for the first time, that rats treated with MVIIA 10 μM 4 h after SCI had increased expression of the anti-apoptotic protein Bcl-xl and reduced expressions of pro-apoptotic Bax, nNOS, caspase-8, and caspase-3. These data corroborated the findings of Fang et al. [98], who observed lower Bax and caspase-3 expressions as well as higher expression of the anti-apoptotic protein after MVIIA injection in a cell-based model of Alzheimer's disease conducted in mice *in vitro* and the same at the cisplatin model *in vitro*, that Leo et al. [59] showed prevented caspase-3 activation. Apoptosis is regulated by biological processes within two main pathways, the extrinsic pathway, which involves ligands that bind to cell death receptors, and the intrinsic pathway, mainly mediated by mitochondria [99]. The Bcl-2 protein family has a central part in controlling the mitochondrial pathway through pro-apoptotic (Bax and Bak) and anti-apoptotic (Bcl-2 and Bcl-xl) proteins [100, 101]. Therefore, balance among these proteins is essential to cell survival or death. MVIIA mitigates Bax expression and augments Bcl-xl levels, to preserve the mitochondrial membrane after injury [102] and to prevent additional ROS production, as observed here. By diminishing ROS production, MVIIA contributes to higher Bcl-xl and lower Bax expressions [103, 104]. By preserving the mitochondria, MVIIA avoids further ROS formation.

Despite the low Bax/Bcl-xl ratio, caspase-9 expression did not decrease in the apoptosis cascade sequence. This could be related to another cascade activation pathway via direct caspase-2 action, as described by Samraj et al. [105], or via mitochondria [106]. Caspase-9 expression peaks lasting hours or days have been reported [107, 108]. However, depending on the spinal cord region and on the experiment, differences may or may not be found at certain evaluation times. Therefore, we suggest that alterations in caspase-9 expression in rats treated with MVIIA may have been due to the assessed time or spinal cord segment. In addition, the important contribution of endoplasmic reticulum stress to caspase-12-mediated apoptotic pathways has been established [109–111]. Caspase-12 expression did not decrease in rats treated with MVIIA 48 h after SCI.

MVIIA reduced the expression of nNOS, a mediator of NO formation. There is strong evidence that NO participates in neuronal death by elevating oxidative stress [112–115] and consequently inducing apoptosis [116–118]. Therefore, by blocking VDCCs, MVIIA might reduce nNOS expression, ROS production, and lipid peroxidation.

The extrinsic caspase-3 activation by caspase-8 elicited by ligands such as TNF-α or Fas L, which are plasma membrane cell death receptors, is another apoptotic pathway [101, 119]. ROS may elevate expression of the cell death receptor Fas L, to trigger caspase-8 action [120, 121]. MVIIA effectively inhibited caspases-8 and -3, which indicated that this pathway was part of this toxin action mechanism. Caspase-8 can also cleave the protein Bid, to augment mitochondrial permeability and to provide communication between the intrinsic and extrinsic pathways [100, 101]. Hence, we suggest that caspase-8 inhibition is related to the higher mitochondrial preservation verified in MVIIA rats.

Together, our results demonstrate for the first time that late intrathecal administration of MVIIA, a blocker of N-type calcium channels, protects spinal cord cells in rats submitted to SCI, to preserve mitochondrial viability and to abate oxidative stress, cell death, and expression of pro-apoptotic factors. Our data also show that MVIIA can positively modulate the antioxidant system and antiapoptotic factors, which makes MVIIA a promising therapy for SCI, especially for the excellent results applied hours after the trauma, which is the greatest challenge for SCI.

## Supporting information

S1 Table Basso, Beattie, and Bresnahan locomotor rating scale was employed in this experiment [44]. (DOCX)

S2 Table Percentage of MVIIA injection side effects after spinal cord injury in Wistar rats. (DOCX)

S1 Fig MVIIA effect on cell death 48 h after spinal cord injury in Wistar rats. Representative images of the ethidium homodimer-stained lateral funiculus of spine slices obtained from Wistar rats submitted to dorsal laminectomy (SHAM rats, negative control) or to spinal cord injury and subsequent injection, 4 h after trauma, of placebo (PBS, PLA rats) or MVIIA (MVIIA 2.5, 5, 10, and 20 μM rats).
(TIFF)

S2 Fig MVIIA effect on cell death 48 h after spinal cord injury in Wistar rats. Representative images of the ethidium homodimer-stained lateral funiculus of spine slices obtained from Wistar rats submitted to dorsal laminectomy (SHAM rats, negative control) or to spinal cord injury and subsequent injection of placebo (PBS, PLA rats) or MVIIA 10 μM one hour or four hours after surgery (MVIIA 10 μM 1h rats or MVIIA 10 μM 4h rats).
(TIFF)
