# Supplementary material for: Conotoxin MVIIA improves cell viability and antioxidant system after spinal cord injury in rats
Source: PLoS One. 2018 Oct 4;13(10):e0204948. doi: 10.1371/journal.pone.0204948 (PMC6171875; doi:10.1371/journal.pone.0204948)
Supplement: S1 Table — (DOCX) [file pone.0204948.s001.docx]

**S1 Table. Basso, Beattie, and Bresnahan Locomotor Rating Scale was employed in this experiment [44].**

| **Score** | **Description** |
| --- | --- |
| 0 | No observable hindlimb movement |
| 1 | Slight movement of one or two joints, usually the hip and/or knee |
| 2 | Extensive movement of one joint or extensive movement of one joint and slight movement of one other joint |
| 3 | Extensive movement of two joints |
| 4 | Slight movement of all three joints of the hindlimb |
| 5 | Slight movement of two joints and extensive movement of the third |
| 6 | Extensive movement of two joints and slight movement of the third |
| 7 | Extensive movement of all three joints of the hindlimb |
| 8 | Sweeping with no weight support or plantar placement of the paw with no weight support |
| 9 | Plantar placement of the paw with weight support in stance only (i.e., when stationary) or occasional, frequent, or consistent weight-supported dorsal stepping and no plantar stepping |
| 10 | Occasional weight-supported plantar steps; no forelimb–hindlimb coordination |
| 11 | Frequent to consistent weight-supported plantar steps and no forelimb–hindlimb coordination |
| 12 | Frequent to consistent weight-supported plantar steps and occasional forelimb–hindlimb coordination |
| 13 | Frequent to consistent weight-supported plantar steps and frequent forelimb–hindlimb coordination |
| 14 | Consistent weight-supported plantar steps, consistent forelimb–hindlimb coordination, and predominant paw position during locomotion is rotated (internally or externally) when it makes initial contact with the surface as well as just before it is lifted off at the end of stance; or frequent plantar stepping, consistent forelimb–hindlimb coordination, and occasional dorsal stepping |
| 15 | Consistent plantar stepping and consistent forelimb–hindlimb coordination and no toe clearance or occasional toe clearance during forward limb advancement; predominant paw position is parallel to the body at initial contact |
| 16 | Consistent plantar stepping and consistent forelimb–hindlimb coordination during gait and toe clearance occurs frequently during forward limb advancement; predominant paw position is parallel at initial contact and rotated at lift off |
| 17 | Consistent plantar stepping and consistent forelimb–hindlimb coordination during gait and toe clearance occurs frequently during forward limb advancement; predominant paw position is parallel at initial contact and lift off |
| 18 | Consistent plantar stepping and consistent forelimb–hindlimb coordination during gait and toe clearance occurs consistently during forward limb advancement; predominant paw position is parallel at initial contact and rotated at lift off |
| 19 | Consistent plantar stepping and consistent forelimb–hindlimb coordination during gait, toe clearance occurs consistently during forward limb advancement, predominant paw position is parallel at initial contact and lift off, and tail is down part or all of the time |
| 20 | Consistent plantar stepping and consistent coordinated gait, consistent toe clearance, predominant paw position is parallel at initial contact and lift off, and trunk instability; tail consistently up |
| 21 | Consistent plantar stepping and coordinated gait, consistent toe clearance, predominant paw position is parallel throughout stance, and consistent trunk stability; tail consistently up |
