# Supplementary material for: Conotoxin MVIIA improves cell viability and antioxidant system after spinal cord injury in rats
Source: PLoS One. 2018 Oct 4;13(10):e0204948. doi: 10.1371/journal.pone.0204948 (PMC6171875; doi:10.1371/journal.pone.0204948)
Supplement: S2 Table — (DOCX) [file pone.0204948.s002.docx]

**S2 Table. Percentage of MVIIA injection side effects after spinal cord injury in Wistar rats.**

| Group | MVIIA side effects (rats/%) |
| --- | --- |
| 2,5 µM | 0/7 (0) |
| 5 µM | 0/7 (0) |
| 10 µM | 0/7 (0) |
| 20 µM | 7/7 (100) |
