# Supplementary figures and images for: Conotoxin MVIIA improves cell viability and antioxidant system after spinal cord injury in rats
Source: PLoS One. 2018 Oct 4;13(10):e0204948. doi: 10.1371/journal.pone.0204948 (PMC6171875; doi:10.1371/journal.pone.0204948)

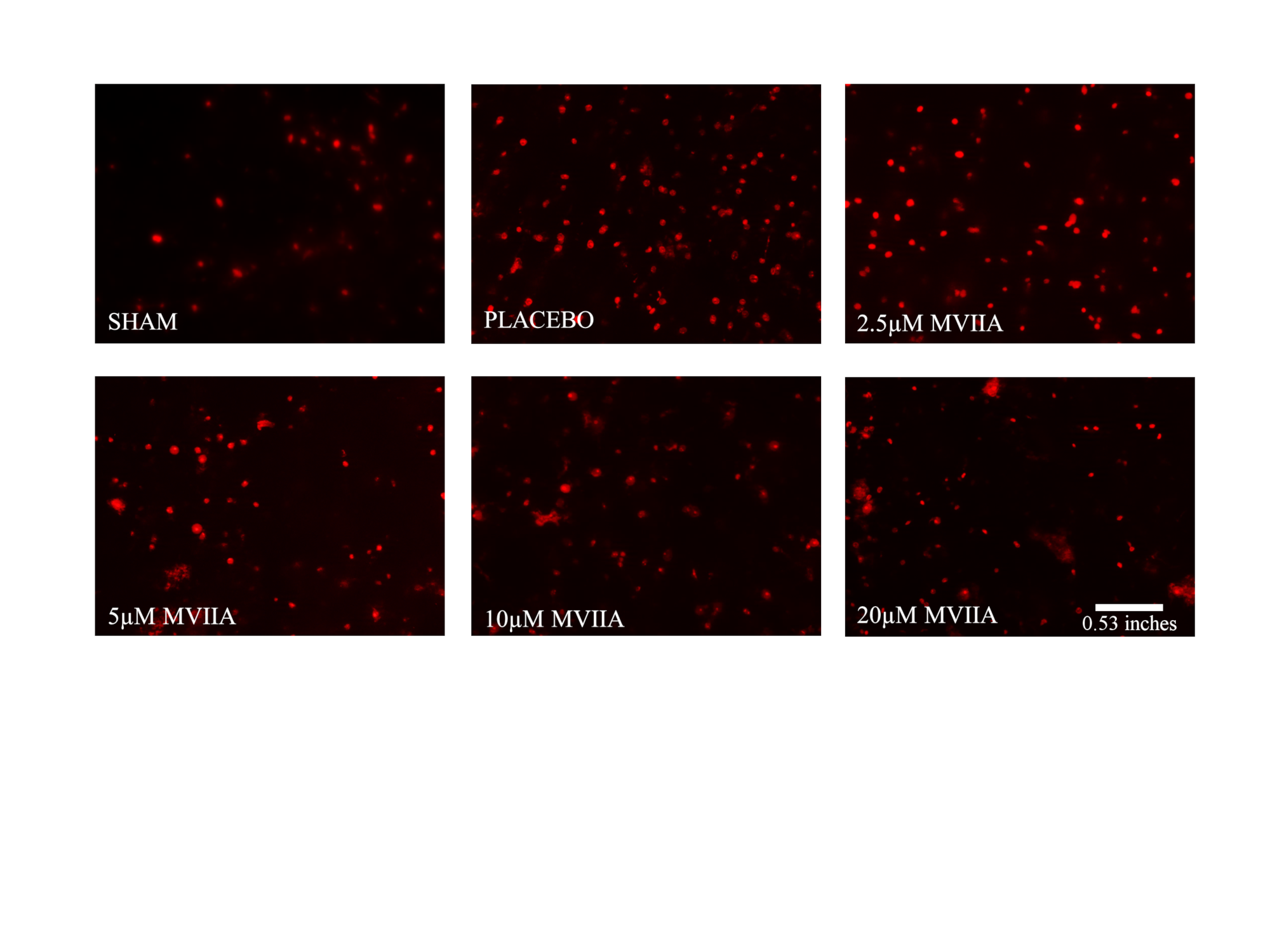

Supplement: S1 Fig — Representative images of the ethidium homodimer-stained lateral funiculus of spine slices obtained from Wistar rats submitted to dorsal laminectomy (SHAM rats, negative control) or to spinal cord injury and subsequent injection, 4 h after trauma, of placebo (PBS, PLA rats) or MVIIA (MVIIA 2.5, 5, 10, and 20 μM rats). (TIFF) [file pone.0204948.s003.tiff]

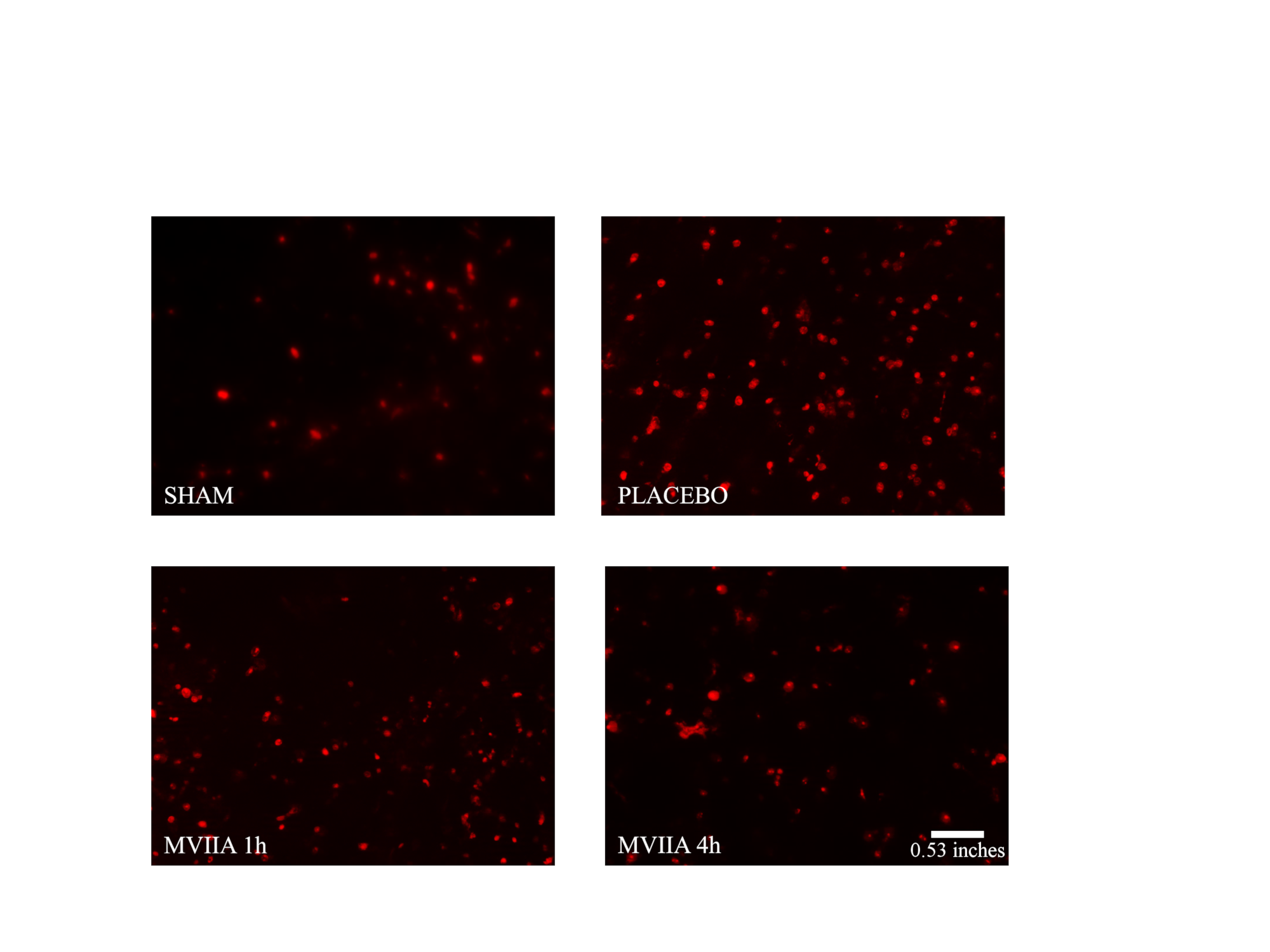

Supplement: S2 Fig — Representative images of the ethidium homodimer-stained lateral funiculus of spine slices obtained from Wistar rats submitted to dorsal laminectomy (SHAM rats, negative control) or to spinal cord injury and subsequent injection of placebo (PBS, PLA rats) or MVIIA 10 μM one hour or four hours after surgery (MVIIA 10 μM 1h rats or MVIIA 10 μM 4h rats). (TIFF) [file pone.0204948.s004.tiff]
